# Real-World Data on Institutional Implementation of Screening for Mismatch Repair Deficiency and Lynch Syndrome in Endometrial Cancer Patients

**DOI:** 10.3390/cancers16030671

**Published:** 2024-02-04

**Authors:** Carmen Joder, Andrea Gmür, Wiebke Solass, Lucine Christe, Manuela Rabaglio, Muriel Fluri, Tilman T. Rau, Flurina A. M. Saner, Laura Knabben, Sara Imboden, Michael D. Mueller, Franziska Siegenthaler

**Affiliations:** 1 Faculty of Medicine, University of Bern, 3010 Bern, Switzerland; carmen.joder@students.unibe.ch; 2 Department of Obstetrics and Gynecology, Bern University Hospital, 3010 Bern, Switzerland; 3 Institute of Tissue Medicine and Pathology, University of Bern, 3010 Bern, Switzerland; 4 Department of Medical Oncology, Bern University Hospital, 3010 Bern, Switzerland; 5 Institute of Pathology, Universitätsklinikum Düsseldorf, 40225 Düsseldorf, Germany

**Keywords:** Lynch syndrome, mismatch repair deficiency, endometrial cancer, testing adherence, genetic counseling, genetic testing, quality control, reflex screening

## Abstract

**Simple Summary:**

Lynch syndrome is one of the most common tumor syndromes and the leading cause of hereditary endometrial cancer. International guidelines recommend universal screening for Lynch syndrome in women with endometrial cancer as they may benefit from personalized cancer treatment, targeted cancer surveillance, and risk-reducing prevention strategies. However, testing for Lynch syndrome is not yet well established in clinical practice, compromising optimal medical care for affected women and their at-risk relatives. In this study, we examine the implementation of Lynch syndrome screening using real-world data. We further analyze intermediate steps of testing for common sources of error and investigate patient risk factors leading to non-adherence to testing. To promote precision healthcare and ensure future quality of care for Lynch syndrome patients, we discuss strategies to optimize the Lynch syndrome screening algorithm in clinical practice.

**Abstract:**

Lynch syndrome is an inherited tumor syndrome caused by a pathogenic germline variant in DNA mismatch repair genes. As the leading cause of hereditary endometrial cancer, international guidelines recommend universal screening in women with endometrial cancer. However, testing for Lynch syndrome is not yet well established in clinical practice. The aim of this study was to evaluate adherence to our Lynch syndrome screening algorithm. A retrospective, single-center cohort study was conducted of all endometrial cancer patients undergoing surgical treatment at the Bern University Hospital, Switzerland, between 2017 and 2022. Adherence to immunohistochemical analysis of mismatch repair status, and, if indicated, to MLH1 promoter hypermethylation and to genetic counseling and testing was assessed. Of all 331 endometrial cancer patients, 102 (30.8%) were mismatch repair-deficient and 3 (0.9%) patients were diagnosed with Lynch syndrome. Overall screening adherence was 78.2%, with a notable improvement over the six years from 61.4% to 90.6%. A major reason for non-adherence was lack of provider recommendation for testing, with advanced patient age as a potential patient risk factor. Simplification of the algorithm through standardized reflex screening was recommended to provide optimal medical care for those affected and to allow for cascading testing of at-risk relatives.

## 1. Introduction

Lynch syndrome is an autosomal dominant genetic disorder, one of the most common inherited tumor syndromes and the leading cause of hereditary colorectal and endometrial cancer [1,2,3]. It is caused by a heterozygous germline mutation in the genes encoding mismatch repair (MMR) proteins, which results in MMR deficiency when combined with a second-hit mutation of the remaining wild-type allele [4,5]. Such a deficiency in DNA repair mechanisms disrupts the integrity of the cellular genome, leading to increased mutational load, microsatellite instability and consequently increased tumor susceptibility [6,7]. Given the increased risk of cancer, early identification of individuals with Lynch syndrome is crucial for optimal clinical management, as they can benefit from personalized cancer treatment, syndrome-specific cancer surveillance, and risk-reducing prevention strategies [8,9,10,11,12,13,14,15]. In addition to the importance of a Lynch syndrome diagnosis for the affected individual, it also has significant implications for their relatives. Due to autosomal dominant inheritance, there is a 50% chance of Lynch syndrome occurring in any first-degree relative, regardless of gender [3]. Therefore, when Lynch syndrome is confirmed, cascade testing should identify those unaffected carriers who may also benefit from syndrome-specific surveillance programs [16,17]. In endometrial cancer, universal screening for Lynch syndrome has recently been recommended by several national and international guidelines [8,18,19]. However, screening for Lynch syndrome in women with endometrial cancer has not yet been adequately implemented in clinical practice [20,21]. As a result, neither optimal care for patients with Lynch syndrome nor comprehensive cascade testing to identify relatives with Lynch syndrome can be achieved. 

To address this issue, our study examined adherence to Lynch syndrome screening in endometrial cancer patients at the University Hospital of Bern and its development over time. To ensure future quality of screening for Lynch syndrome in endometrial cancer patients, interim steps of testing were reviewed for common sources of error and patient risk factors for non-adherence were identified.

## 2. Materials and Methods

### 2.1. Patient Population

A retrospective observational study was conducted, including all patients with endometrial cancer who underwent surgical treatment at the University Hospital of Bern, Switzerland, between 2017 and 2022. Inclusion criteria were age over 18 years and first manifestation of histologically confirmed endometrial carcinoma. All patients provided general consent for their data to be used for research purposes. Clinical and histopathological data were retrieved from a central electronic database.

### 2.2. Immunohistochemistry, Methylation Analysis, and Molecular–Genetic Testing

**Figure 1:**
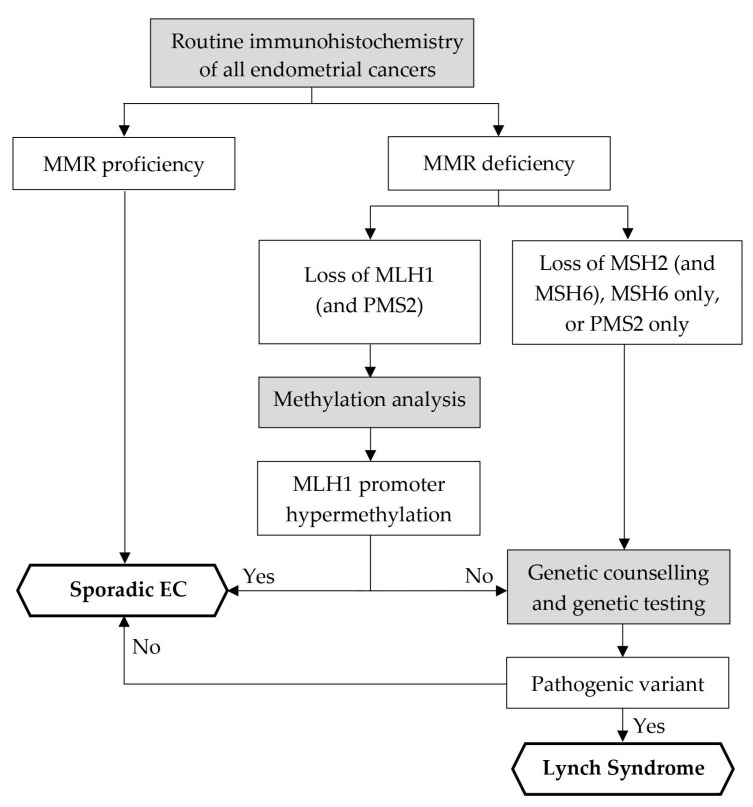
Flowchart of Lynch syndrome screening algorithm at our institution. In accordance with the recommendations of the Manchester International Consensus Group [8] and AWMF [18]. Abbreviations: *MMR*—mismatch repair; *EC*—endometrial cancer.

Mismatch repair status was determined by immunohistochemistry at the time of endometrial biopsy or at the time of surgical resection. A four-antibody testing algorithm consisting of antibodies for MLH1 (RTU-Predilute, clone M1, Roche Diagnostics), MSH2 (RTU-Predilute, clone G219-1129, Roche Diagnostics), MSH6 (RTU-Predilute, clone SP93, Roche Diagnostics), and PMS2 (RTU-Predilute, clone A16-4, Roche Diagnostics) was applied using the VENTANA BenchMark ULTRA platform (Roche Diagnostics) and OptiView DAB IHC v6 (Roche Diagnostics) as DetectionKit. Depending on the expression profile of MMR proteins in immunohistochemistry, the patient population was divided into two cohorts: MMR-proficient (MMRp), if all four proteins showed normal expression, and MMR-deficient (MMRd), if at least one of the above-mentioned MMR proteins showed aberrant expression. MLH1-deficient cancer tissue was subsequently analyzed for MLH1 promoter hypermethylation at the request of the treating physician. In MLH1 promoter methylation analysis, the MLH1 promoter region was amplified by PCR after bisulfite modification of tumor DNA and then analyzed for hypermethylation by pyrosequencing. This method allows for the detection of 5 potentially methylated CpG dinucleotides located in the promoter region of the MLH1 gene. Hypermethylation was defined as >10% methylation in at least 4 of 5 CpG dinucleotides analyzed. The minimum allele frequency to detect a hypermethylation was 10% [22]. All MLH1-deficient endometrial carcinomas with MLH1 promoter hypermethylation were considered sporadic, excluding the possibility of the presence of a pathological germline mutation and thus Lynch syndrome. Cases with protein expression loss of MLH1 without MLH1 promoter hypermethylation, loss of PMS2 only, or loss of MSH2 and/or MSH6 were triaged as potential Lynch syndrome-associated MMRd endometrial cancer. Recommendation for genetic counseling and testing was subsequently made at multidisciplinary tumor board meetings and registered by the treating physician. Genetic counseling was provided by certified counsellors from the University Hospital of Bern and genetic testing was subsequently performed in accordance with the patient’s informed consent. For genetic screening, sequence analysis of the coding exons (including exon/intron boundaries, +/−10 base pairs) of the Lynch syndrome-associated mismatch repair genes *MLH1*, *MSH2*, *MSH6*, and *PMS2* was performed by high-throughput sequencing, Sanger sequencing of selected exons, and gene dosage analyses by MLPA assays. If no deleterious sequence variant was found in *MSH2* despite immunohistochemical loss of MSH2 and/or MSH6, the *EPCAM* gene was analyzed as its deletion can lead to methylation of the *MSH2* promoter, resulting in silencing of the *MSH2* gene [4]. Lynch syndrome was diagnosed in all patients with a confirmed pathogenic or likely pathogenic MMR gene variant. Figure 1 demonstrates the flow chart of the screening algorithm applied at our center.

### 2.3. Workup and Management of Patients after Diagnosis of Lynch Syndrome

The work-up and management of patients with Lynch syndrome at our institution follows national and international guidelines and consists of syndrome-specific surveillance and prevention strategies [18,23]. Given the well-documented survival benefit for patients with Lynch syndrome with regular colorectal cancer surveillance, colonoscopy is recommended every 1–2 years from the age of 25. In addition, upper gastrointestinal endoscopy is recommended every 2–4 years from the age of 30–40, including Helicobacter screening and eradication therapy if necessary. For gynecological surveillance of patients with Lynch syndrome, annual gynecological examination is recommended, including transvaginal ultrasound with endometrial biopsies every 1–2 years from age 30–35. In addition, a better understanding of early symptoms of gynecologic cancers is encouraged in Lynch syndrome patients, as this is crucial to improve early diagnosis [8]. Depending on the affected gene and the associated tumor risks, further surveillance strategies are recommended. Considering family planning, menopausal status, and the affected gene, bilateral salpingo-oophorectomy and hysterectomy may be considered as risk-reducing surgeries and will be discussed individually. Patients are advised to inform other family members about the hereditary nature of Lynch syndrome and the possibility of pre-symptomatic genetic testing. Cascade testing of relatives is targeted as early detection of a genetic mutation allows surveillance and prevention strategies to be implemented at an earlier age [16,17].

### 2.4. Statistical Analysis 

Statistical analysis was performed using the Statistical Package for Social Sciences (IBM SPSS Statistic version 28.0.1.1). Continuous variables were reported as means and standard deviations (±SD), while categorical variables were reported as frequencies and proportions (%). Formal comparisons were made using analysis of variance (ANOVA) for continuous variables and chi-square statistics for categorical variables. Statistical significance was defined as a *p*-value below 0.05.

Adherence to the Lynch syndrome screening algorithm, consisting of immunohistochemistry and, if indicated, promoter methylation analysis, genetic counseling, and genetic testing, was considered correct if all the interim steps shown in Figure 1 were performed as required. Adherence to MMR status testing was considered correct if MMR status was determined by immunohistochemistry in all newly diagnosed endometrial cancers. Adherence to methylation analysis was considered correct if MLH1 promoter methylation status was determined in all patients with aberrant MLH1 protein expression. Adherence to genetic counseling and testing was considered correct if a patient with loss of protein expression of PMS2 only, MSH2 and/or MSH6, or MLH1/PMS2 without MLH1 promoter hypermethylation received genetic counseling and testing. If the patient was offered genetic testing but declined it, adherence to genetic testing was considered correct.

## 3. Results

### 3.1. Study Population

A total of 331 patients met the inclusion criteria and were enrolled in the present study. At initial diagnosis, the mean age of the study population was 65.6 (SD ± 11.4) years, and mean BMI was 29.2 (SD ± 7.8) kg/m^2^. MMR status was defined in 307 (92.7%) patients and missing in 24 (7.3%) patients. A total of 205 (61.9%) patients presented with mismatch repair proficiency (MMRp) and 102 (30.8%) patients with mismatch repair deficiency (MMRd) (Table 1). Of all MMRd patients, seventy-nine (77.5%) patients presented with MLH1/PMS2 loss, eleven (10.8%) with loss of MSH6 only, seven (6.9%) with MSH2/MSH6 loss, four (3.9%) with loss of PMS2 only, and one (1%) with MLH1/MSH2 loss. After immunohistochemistry and methylation analysis, 24 patients were triaged as potential Lynch syndrome-associated, showing loss of expression of the MMR proteins PMS2 only (N = 4), MSH2/MSH6 (N = 7), MSH6 only (N = 11), MLH1/MSH2 (N = 1), and MLH1/PMS2 without MLH1 promoter hypermethylation (N = 1). A total of three germline pathogene_MMR variant carriers were identified, accounting for a Lynch syndrome prevalence of 0.9% in the overall study cohort and 2.9% within the MMRd cohort. There was one carrier each of the variant path_MSH2, path_PMS2, and path_MSH6. Patients with Lynch syndrome-related endometrial cancer had a mean age of 52.7 (SD ± 5.5) years at initial diagnosis and a mean BMI of 23.5 (SD ± 3.3) kg/m^2^. There was no positive personal history of cancer in any of the three Lynch syndrome cases, making endometrial cancer the first manifestation of malignancy. Two of the three women with confirmed Lynch syndrome were managed as described (2.3 Workup and Management of Patients after Diagnosis of Lynch Syndrome), while one woman refused any further workup after diagnosis. 

### 3.2. Adherence to Immunohistochemistry, Methylation Analysis, and Genetic Testing

**Figure 2:**
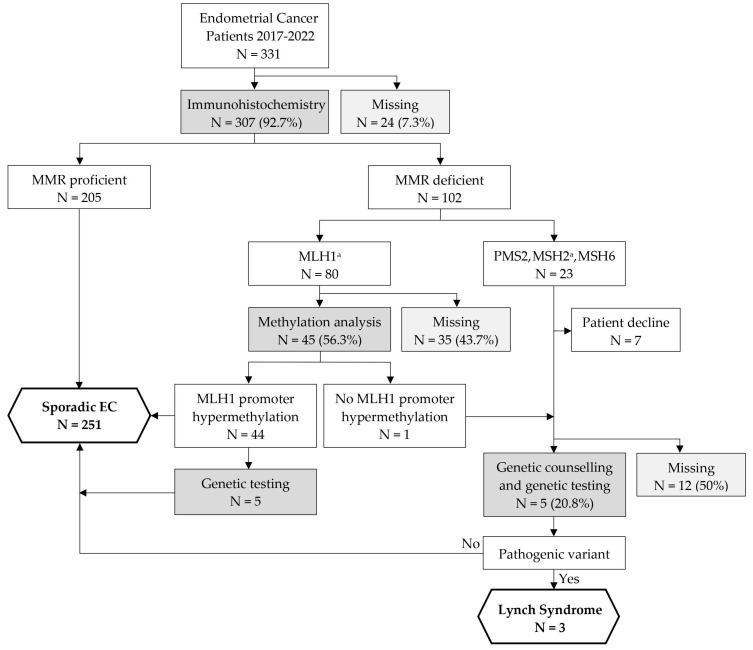
Flowchart of adherence to Lynch syndrome screening algorithm at our institution. ^a^ MLH1/MSH2 loss was classified as both MLH1 and MSH2 loss. Abbreviations: N—number; MMR—mismatch repair; EC—endometrial cancer.

Adherence to the Lynch syndrome screening algorithm was assessed over the six-year study period. The results are shown in Figure 2. Implementation of the screening algorithm during the study period was correct in 259 (78.2%) of all 331 patients in the study cohort and in 55 (53.9%) of 102 patients with MMRd endometrial cancer. In 307 (92.7%) endometrial cancer patients, MMR status was determined by immunohistochemistry. In 24 (7.3%) patients, immunohistochemistry was not performed for unknown reasons, resulting in an adherence rate of 92.7% for MMR status testing. Of all 80 patients with loss of MLH1 protein expression, testing for MLH1 promoter methylation status was suggested in 51 (63.8%) and performed in 45 (56.3%) patients. Only one (2.2%) of the tested patients presented with MLH1 deficiency without MLH1 promoter hypermethylation, whereas forty-four (97.8%) patients had MLH1-hypermethylated MMRd endometrial cancer. Overall, MLH1 promoter methylation status was correctly assessed in 45 of 80 patients, resulting in a methylation analysis adherence rate of 56.3%. Among the 102 MMRd patients, genetic counseling was suggested in 30 (29.4%) and performed in 12 (11.8%) patients, while genetic testing was performed in 10 (9.8%) patients. The reasons for not performing genetic testing in 92 of 102 MMRd patients were lack of indication due to MLH1 promoter hypermethylation in 39 (42.4%), patient decline in 7 (7.6%), age in 9 (9.8%), and unknown in 37 (40.2%) cases. Of the ten patients who underwent genetic testing, five were genetically tested even though they had MLH1 promoter hypermethylation, but none of them were carriers of a germline pathogene_MMR variant. Of the 24 patients with potentially Lynch syndrome-associated endometrial cancer, genetic counseling was suggested in 17 (70.8%) patients. Five (20.8%) received genetic testing and seven (29.2%) declined, leaving an additional twelve (50%) patients who should have received genetic testing. Of the five patients genetically tested, three tested positive for Lynch syndrome.

### 3.3. Testing Adherence over Time

**Figure 3:**
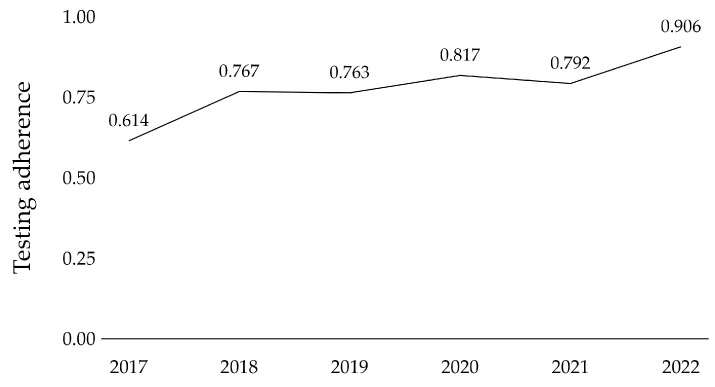
Development of testing adherence over the course of 2017 to 2022 in the patient cohort.

Trends in overall adherence to the Lynch syndrome screening algorithm were further analyzed from 2017 to 2022 and are presented in Figure 3. Testing adherence improved significantly over the six-year study period for the entire patient cohort, from 61.4% to 90.6% (*p* = 0.026). In the last year of the study period, overall adherence to screening was 90.6%, with 100% adherence to MMR immunohistochemistry, 81.2% to MLH1 promoter methylation analysis, and 73.7% to genetic counseling and testing.

### 3.4. Potential Sources of Error in the Follow-Up Screening of Endometrial Cancer Patients with Mismatch Repair Deficiency 

Twenty-nine out of thirty-five patients with missing MLH1 promoter methylation analysis and seven out of twelve patients with missing genetic testing were incorrectly screened due to lack of recommendation. The main reason for non-adherence to the Lynch syndrome screening algorithm was therefore a missing test recommendation by the treating physician.

The underlying causes of incorrect follow-up of MMR-deficient endometrial cancer patients in relation to the Lynch syndrome screening algorithm were analyzed and presented in Table 2. A significant correlation was found between lower adherence to testing and older patient age (*p* < 0.001). Regarding tumor stage, patients with advanced-stage tumors tended to be more prone to non-adherence than patients with early-stage tumors, although this was statistically not significant. In terms of patient counseling, there was a trend that patients who had their follow-up control by a gynecological oncologist were more likely to be tested correctly than patients who were counseled by a general gynecologist.

## 4. Discussion

### 4.1. Main Findings

In the present study, we evaluated the clinical adherence to the Lynch syndrome screening algorithm at Bern University Hospital over a study period of six years. Adherence to screening was 78.2% for all patients with endometrial cancer and 53.9% for all patients with MMRd endometrial cancer, indicating that despite national guideline recommendations, there is a considerable missing rate in the detection of Lynch syndrome in endometrial cancer patients. Over the past six years, our data have shown a remarkable improvement in adherence to testing. In the final year, overall adherence to the Lynch syndrome screening algorithm improved to 90.6% of all endometrial cancer patients, which is consistent with previous findings [24,25]. However, while immunohistochemical testing for MMR status was correctly performed in all patients, almost one-fifth (19.8%) of all MLH1-deficient patients did not undergo MLH1 promoter methylation testing and approximately one quarter (26.3%) of all patients suspected of having Lynch syndrome did not undergo genetic testing. In accordance with recent findings, the recommendation and implementation of genetic counseling and testing proved to be particularly error-prone and in need of improvement [24,26,27,28]. Previous studies have shown that only about 60% of high-risk women with endometrial cancer are referred for genetic counseling, and only 35–40% undergo genetic testing [27,28]. In addition to several previously described barriers to follow-up genetic counseling and testing, such as lack of clinician knowledge, cultural barriers, cost, lack of insurance, travel time, and timing of re-consultation, patient decline has been identified as an important reason for not screening for Lynch syndrome [21,24,27,28,29]. Our data showed that 29.2% of all patients suspected of having Lynch syndrome declined genetic counseling and one patient diagnosed with Lynch syndrome declined further follow-up. Possible reasons for patient refusal include lack of knowledge about the possible consequences of a test result, underestimation of the personal risk of Lynch syndrome-related hereditary cancer and the risks for the family members, general resistance to genetic testing, and fear and anxiety about test results [27,28,30]. Apart from patient refusal, the reasons for non-adherence to the screening algorithm in our study population are unknown in most cases. However, our data suggest a high error rate due to lack of test recommendation by the attending physician, confirming the findings of Lentz et al., who showed that the requirement for a physician’s recommendation resulted in a significant missing rate of women to be screened, and thus reduced efficiency of care [25]. Advanced patient age was found to be a significant reason for not recommending and therefore not correctly following up MMRd patients. This is consistent with previous findings that women with cancer at an earlier stage and at a younger age were more likely to be referred for genetic counseling [31]. Although patients with Lynch syndrome develop endometrial cancer at a younger age on average, several data have shown that a screening algorithm with an upper age limit would result in a significant detection loss of Lynch syndrome cases [32,33]. It has also been shown that universal screening for Lynch syndrome is more cost-effective than limiting screening based on age or family history [34]. Further, it should be noted that even if a patient of advanced age and tumor stage with a poor prognosis is unlikely to benefit from a diagnosis of Lynch syndrome, the test should still be performed as the diagnosis of Lynch syndrome has implications for family members who may be affected [16,17]. Recent guidelines therefore recommend screening without an upper age limit, indicating that high patient age should no longer be a reason for not testing [8].

### 4.2. Future Directions

The identification of individuals with Lynch syndrome is essential for providing appropriate medical care, as patients and their families can benefit from genetic counseling regarding additional cancer risks, specific surveillance programs, and cancer prevention strategies [8,9,10,11,12,13,14,15]. However, as the implementation of the Lynch syndrome screening algorithm remained improvable in the past year, we recommend the introduction of a standardized screening procedure to optimize testing adherence and thereby ensure appropriate clinical management of endometrial cancer patients at risk of Lynch syndrome. We hereby recommend immunohistochemical testing for MMR status in all patients with newly diagnosed endometrial cancer, regardless of age. If MMR status is inconclusive after external examination of MMR proteins, repeat immunohistochemistry is required on referral of the patient. For all patients with loss of MLH1 protein expression, promoter methylation analysis should be performed automatically by the pathology department, without the need for testing to be requested by the attending physician. This reflex methylation analysis eliminates the high error rate due to lack of provider recommendation and reduces the burden on ordering physicians [25]. Following MMR immunohistochemistry and MLH1 promoter methylation analysis, all cases with loss of protein expression of PMS2 only, MSH2 and/or MSH6, or MLH1 without MLH1 promoter hypermethylation need to be triaged as potential Lynch syndrome-associated endometrial cancer. All such cases should be referred directly for genetic counseling within the scope of the multidisciplinary tumor board recommendation, with effective communication between healthcare professionals and a clear assignment of responsibilities being essential. In addition, the awareness and willingness of gynecologists to screen for Lynch syndrome needs to be increased and additional training may be required [26]. Genetic testing must be performed after genetic counseling and with the patient’s general consent. To minimize patient refusal of genetic counseling, patient health literacy, awareness, and understanding of Lynch syndrome need to be improved. Effective patient education is therefore required prior to genetic counseling [27]. If Lynch syndrome is diagnosed, further information should be provided to the patient by the genetic counsellor and cascade testing should be attempted in all family members at risk. This simplification of the screening algorithm through standardized reflex screening, clearly defined roles and responsibilities, and improved patient and physician education could reduce the possibility of human error and contribute to a quality improvement in the clinical management of endometrial cancer patients, thereby promoting precision healthcare [35].

An additional issue to be discussed for future directions in clinical screening for Lynch syndrome is the possibility of MLH1 hypermethylation in Lynch syndrome patients who are carriers of a pathogenic MLH1 germline variant [36,37,38] or who are carriers of a constitutional, possibly hereditary, MLH1 epimutation [36,39,40,41]. The co-occurrence of a somatic MLH1 hypermethylation with a germline MLH1 mutation has been described in a proportion of more than 15%, indicating that Lynch syndrome cannot be excluded when MLH1 promoter hypermethylation is observed [36]. In addition, individual cases of constitutional MLH1 methylation have been described as a rare cause of Lynch syndrome [36,39,40,41]. These findings suggest that MLH1 hypermethylation is not an exclusive mechanism of non-inherited cancers and that it plays a non-negligible role in Lynch syndrome-related cancers [37]. Therefore, genetic testing for a pathogenic MLH1 variant as well as screening for constitutional MLH1 epimutations and promoter sequence alterations in MLH1-hypermethylated tumors in the context of the patient’s family and personal history have been discussed [37,39,40]. However, future studies are needed to elaborate the implications of these findings for clinical screening for Lynch syndrome in women with MLH1-hypermethylated endometrial cancer.

### 4.3. Strength and Limitations 

In our opinion, the main strengths of this study are the long time period and the large sample size, which allowed us to thoroughly investigate adherence to the Lynch syndrome screening algorithm. Another strength is the detailed assessment of adherence to all intermediate steps, which allows for a more accurate identification of potential sources of error. The main limitations of this study are its single-center design and its retrospective nature. By collecting data retrospectively from a central electronic database, our data were highly dependent on the quality of data documentation. This exposed our data to potential bias, as any undocumented steps were considered non-existent and may have led to an underestimation of test adherence. Nevertheless, this study highlights the importance of quality control in clinical practice. Analysis of our data identified common sources of error and provided recommendations to improve adherence to the Lynch syndrome screening algorithm, with the goal of providing evidence-based, high-quality medical care to women with endometrial cancer.

## 5. Conclusions

Despite a significant improvement in adherence to the Lynch syndrome screening algorithm in endometrial cancer patients over the study period, the clinical implementation of screening at our institution is not yet sufficient and should be further optimized. A major reason for non-adherence was a lack of provider recommendation, with advanced patient age as a potential patient risk factor. As the identification of patients with Lynch syndrome is important not only for optimal medical care of the affected individuals, but also for cascade testing of all at-risk relatives, an error-free algorithm for Lynch syndrome screening should be aimed for.

## Figures and Tables

**Table 1:** Mismatch repair status of the study cohort.

MMR Status	N = 331 (%)
MMR-proficient	205 (61.9)
MMR-deficient	102 (30.8)
MLH1/PMS2	79 (23.9)
PMS2 only	4 (1.2)
MSH2/MSH6	7 (2.1)
MSH6 only	11 (3.3)
MLH1/MSH2	1 (0.3)
Unknown MMR status	24 (7.3)

Abbreviations: N—number; MMR—mismatch repair.

**Table 2:** Potential causes of incorrect follow-up screening of MMR-deficient endometrial cancer patients.

	Correct Follow-up in Case of MMRd N = 55	Incorrect Follow-up in Case of MMRd N = 47	*p*-Value ^a^
Mean age ± SD, y	62.9 ± 11.7	71.4 ± 12.4	**<0.001**
Mean BMI ± SD, kg/m^2^	28.2 ± 5.9	28.4 ± 6.2	0.848
Family history, N (%)			0.780
Positive	12 (21.8)	9 (19.1)	
Negative	37 (67.3)	32 (68.1)	
Missing	6 (10.9)	6 (12.8)	
Tumor stage, N (%)			0.062
Early stage	47 (85.5)	33 (70.2)	
Late stage	8 (14.5)	14 (29.8)	
Patient counseling and			0.087
follow-up control, N (%)			
Gynecologic oncologist	11 (20)	4 (8.5)	
General gynecologist	42 (76.4)	43 (91.5)	
Not assessed	2 (3.6)	0 (0)	

^a^ *p* values reflect χ^2^ statistics for categorical variables and ANOVA for age and BMI. A statistically significant *p*-value lower than 0.05 is marked in bold in the table. Early stage = FIGO I + II, late stage = FIGO III + IV. Abbreviations: N—number; SD—standard deviation; y—years; BMI—body mass index.

## Data Availability

The data presented in this study are available from the corresponding author upon request.
